# Newly emerged resistance-breaking variants of cucumber mosaic virus represent ongoing host-interactive evolution of an RNA virus

**DOI:** 10.1093/ve/veaa070

**Published:** 2020-11-07

**Authors:** Kyeong-Jae Heo, Sun-Jung Kwon, Mi-Kyeong Kim, Hae-Ryun Kwak, Soo-Jung Han, Min-Jun Kwon, A L N Rao, Jang-Kyun Seo

**Affiliations:** Department of International Agricultural Technology; Institutes of Green Bio Science and Technology, Seoul National University, 1447 Pyeongchang-ro, Pyeongchang 25354, Republic of Korea; Department of Plant Medicine, Chungbuk National University, 1 Chungdae-ro, Cheongju 28644, Republic of Korea; Crop Protection Division, National Institute of Agricultural Sciences, Rural Development Administration, 300 Nongsaengmyeong-ro, Wanju 55365, Republic of Korea; Department of International Agricultural Technology; Department of International Agricultural Technology; Department of Microbiology and Plant Pathology, University of California, Boyce Hall 1463, 900 University Ave, Riverside, CA 92521, USA; Department of International Agricultural Technology; Institutes of Green Bio Science and Technology, Seoul National University, 1447 Pyeongchang-ro, Pyeongchang 25354, Republic of Korea

**Keywords:** cucumber mosaic virus, RNA virus, evolution, resistance-breaking, pepper

## Abstract

Understanding the evolutionary history of a virus and the mechanisms influencing the direction of its evolution is essential for the development of more durable strategies to control the virus in crop fields. While the deployment of host resistance in crops is the most efficient means to control various viruses, host resistance itself can act as strong selective pressure and thus play a critical role in the evolution of virus virulence. Cucumber mosaic virus (CMV), a plant RNA virus with high evolutionary capacity, has caused endemic disease in various crops worldwide, including pepper (*Capsicum annuum* L.), because of frequent emergence of resistance-breaking variants. In this study, we examined the molecular and evolutionary characteristics of recently emerged, resistance-breaking CMV variants infecting pepper. Our population genetics analysis revealed that the high divergence capacity of CMV RNA1 might have played an essential role in the host-interactive evolution of CMV and in shaping the CMV population structure in pepper. We also demonstrated that nonsynonymous mutations in RNA1 encoding the 1a protein enabled CMV to overcome the deployed resistance in pepper. Our findings suggest that resistance-driven selective pressures on RNA1 might have contributed in shaping the unique evolutionary pattern of CMV in pepper. Therefore, deployment of a single resistance gene may reduce resistance durability against CMV and more integrated approaches are warranted for successful control of CMV in pepper.

## 1. Introduction

Analyzing the genetic diversity and population structure of a virus is an essential approach for understanding its evolutionary history and related mechanisms that drive its evolution and dispersion. Since viruses are obligate intracellular parasites and depend on their hosts for most aspects of the life cycle, they have thus evolved under host-interactive constraints (Roossinck 2003; Lauring et al. 2013). RNA viruses, the largest group of plant viruses, are known to have a rapid evolutionary rate due to error-prone replication and short generation times, allowing for fast virulence changes to sustain infection (Cabanillas et al. 2013; Lauring et al. 2013). In this sense, the widespread use of resistant cultivars may apply significant selective pressures to direct the adaptive virulence evolution of viruses in crop fields (Garcia-Andres et al. 2009). However, mechanistic links between diversity, virulence, and *in vivo* selective pressures are little understood in the crop fields.

*Cucumber mosaic virus* (CMV; genus *Cucumovirus*, family *Bromoviridae*) is one of the most successful RNA viruses for host adaptation and dispersion. CMV has evolved to infect more than 1,200 species, comprising more than eighty plant families and has been dispersed worldwide (Palukaitis and Garcia-Arenal 2003). In over 100 years since its discovery (Doolittle 1916; Jagger 1916), numerous CMV strains and isolates have been identified from various plant species, including dicots and monocots (Jacquemond 2012). Thus far, complete genome sequences of more than 110 CMV isolates have been reported, and extensive analyses of CMV population genetics have been performed to examine the evolutionary history of the virus (Roossinck 2002; Kim et al. 2014; Ohshima et al. 2016). In particular, a previous study showed that CMV populations infecting pepper display unique patterns of evolution in Korea (Kim et al. 2014), suggesting the possibility of the host-adaptive evolution of CMV in pepper. CMV isolates can be divided into three major subgroups (IA, IB, and II), based on their serological and molecular characteristics (Roossinck 2002; Jacquemond 2012). CMV isolates can also be classified into different pathotypes based on their virulence in specific plant species and varieties (Lin et al. 2003; Diveki et al. 2004; Lee et al. 2009).

The CMV genome is divided into three single-stranded RNAs, designated RNA1, RNA2, and RNA3 (Palukaitis and Garcia-Arenal 2003; Jacquemond 2012). RNA1 encodes the 1a protein, which contains two functional domains: an N-terminal methyltransferase domain and a C-terminal helicase domain. RNA2 encodes the 2a protein, which contains a viral RNA-dependent RNA polymerase (RdRp) domain. The 1a and 2a proteins, along with some host factors, comprise the replicase complex. RNA2 also encodes the 2b protein, which has RNA silencing suppressor activity. RNA3 also encodes two proteins, the 3a protein (movement protein; MP) and the coat protein (CP), which are essential for virus movement and transmission. In resistance responses, CMV 2a elicits the hypersensitive response by interacting with the *RT4-4* gene in common bean and with the *Cry* gene in cowpea, while the CMV CP confers the extreme resistance by interacting with the *RCY1* gene in *Arabidopsis thaliana* (Kim and Palukaitis 1997; Takahashi et al. 2002; Seo et al. 2006; Sekine et al. 2008). In pepper, CMV 1a was identified as an avirulence factor that triggers the extreme resistance by interacting with the *Cmr1* gene (Kang et al. 2010, 2012).

CMV is an agriculturally important virus, in addition to being useful to the molecular understanding of RNA viruses. In particular, CMV causes endemic disease in various crops, including pepper (*Capsicum annuum* L.) (Jacquemond 2012; Kim et al. 2014). Although various resistant pepper cultivars have been developed, resistance-breaking variants have continuously emerged, and CMV remains the prevalent virus in pepper crops in Korea and other parts of the world (Ben Tamarzizt et al. 2013; Kim et al. 2014; Choi et al. 2015; Kwon et al. 2018). In this study, we isolated several CMV variants from pepper cultivars resistant to the previous epidemic CMV pathotypes, P0 and P1, classified according to their virulence in indicator pepper cultivars (Lee et al. 2009). Since virulence analysis showed that these collected isolates are highly virulent resistance-breaking variants, we here aimed to investigate the molecular and evolutionary characteristics of the emerged CMV resistance-breaking variants in the CMV population in order to understand the resistance-breaking mechanism. We also performed molecular analyses to examine mechanistic links between CMV evolution and host-associated constraints. Further, we utilized infectious cDNA clones of two CMV strains, CMV-GTN (a resistance-breaking strain isolated from pepper in 2013) and CMV-P1 (a strain isolated from pepper in 2004 belonging to the P1 pathotype), to identify mutations responsible for resistance breaking in pepper (Kang et al. 2012; Choi et al. 2015).

## 2. Materials and methods

### 2.1 Virus sources and plant materials

CMV-GTN was isolated from pepper in our previous study (Choi et al. 2015) and maintained in the pepper cultivar Chungyang. Six additional CMV isolates were collected in this study from pepper cultivars resistant to the CMV P1 pathotype in Korean commercial pepper fields in 2016. The CMV isolates were biologically isolated by mechanical inoculation on the leaves of a local lesion host, *Chenopodium quinoa*. Full-genome sequencing of the CMV isolates was performed as described in our previous study (Kim et al. 2014). Sequences were deposited in the GenBank database (accession numbers are listed in Table 1). Full-length infectious cDNA clones of CMV-Fny and CMV-P1 (pCMV-Fny and pCMV-P1, respectively), generated in our previous studies (Seo et al. 2009a; Kang et al. 2012), were used as viral sources for each strain. CMV-Fny and -P1 were maintained in *Nicotiana benthamiana* plants. Virulence of the CMV strains and isolates was examined in various pepper cultivars, including Baerota, Manita, PR-Sagslee, Quarri, Sinhong, and Superior. All plants inoculated with CMV were grown in an insect-free growth chamber at 25°C under a 16/8-h photoperiod.

**Table 1.** Virulence of CMV strains and isolates in various pepper cultivars.

Isolate or strain	Disease response to CMV inoculation^a^							
	*Nicotiana benthamiana*	*N.tabacum*	Pepper cultivars					
			Sinhong	Quarri	Manita	Superior	Baerota	PR-Sagslee
Fny	S	S	S	S	R	R	R	R
P1	S	S	S	S	S	S	R	R
GTN	S	S	S	S	S	S	S	S
RB1	S	S	S	S	S	S	S	S
RB2	S	S	S	S	S	S	S	S
RB3	S	S	S	S	S	S	S	S
RB4	S	S	S	S	S	S	S	S
RB5	S	S	S	S	S	S	S	S
RB6	S	S	S	S	S	S	S	S

a S, susceptible (systemic mosaic and stunting); R, resistant; CMV infection was verified by RT-PCR analysis using total RNA isolated from upper non-inoculated leaves. Results were obtained from three independent experiments using at least three plants per experiment.

### 2.2 Construction of infectious cDNA clones of CMV-GTN

Total RNA was isolated from pepper leaves infected with CMV-GTN using TRIzol (Invitrogen, Carlsbad, CA, USA) according to the manufacturer’s instructions, and employed for cDNA synthesis of CMV-GTN viral RNA. Specific primers containing the appropriate restriction sites for cloning were designed for amplification of full-length sequences of CMV-GTN RNA1 (GTN-R1-5E-KpnI-Fw: 5′-GGGGTACCGTTTATTTACAAGAGCGTACGG-3′ and GTN-R1R3-3E-BamHI-Rv: 5′-CGGGATCCTGGTCTCCTTTGAGAGACCCC-3′, restriction enzyme sites are underlined), RNA2 (GTN-R2-5E-XbaI-Fw: GCTCTAGAGTTTATTTACAAGAGCGTACGG-3′ and GTN-R2-3E-BamHI-Rv: CGGGATCCTGGTCTCCTTCAGGAAGCCC-3′, restriction enzyme sites are underlined), and RNA3 (GTN-R3-5E-XbaI-Fw: 5′-GCTCTAGAGTAATCTTACCACTGTGTGTGT-3′ and GTN-R1R3-3E-BamHI-Rv: 5′-CGGGATCCTGGTCTCCTTTGAGAGACCCC-3′, restriction enzyme sites are underlined). cDNAs of RNA1, RNA2, and RNA3 of CMV-GTN were synthesized using SuperScript III reverse transcriptase (Invitrogen). The resulting cDNAs were used to amplify full-length RNA1, RNA2, and RNA3 using Q5 DNA polymerase (New England Biolabs, Ipswich, MA, USA) and the primer pairs. Amplified full-length RNA1 was digested with *Kpn*I and *Bam*HI and cloned into the T-DNA region of a modified binary vector, pCassRz (Kwak et al. 2016), opened with *Kpn*I and *Bam*HI. Amplified full-length RNA2 and RNA3 were digested with *Xba*I and *Bam*HI and cloned into the pCassRz vector opened with *Xab*I and *Bam*HI. The resulting constructs were designated pCMV-GTN-R1, -R2, and -R3. The full sequences of the cloned CMV-GTN RNAs were determined by Sanger DNA sequencing and deposited in the GenBank database under accession numbers MN422336 (RNA1), MN422337 (RNA2), and MN422338 (RNA3). The constructs pCMV-GTN-R1, -R2, and -R3 were transformed into *Agrobacterium tumefaciens* strain GV3101.

### 2.3 Generation of chimeric RNA1 constructs between CMV-GTN and -P1

The 1a proteins of pCMV-GTN and -P1 differs only in their amino acids at positions 253 and 553 (nucleotide positions 852 and 1752 in RNA1, respectively), therefore two chimeric RNA1 constructs were generated by exchanging corresponding fragments utilizing three available restriction enzyme sites (*Sal*I site at nucleotide position 577 in RNA1, *Fsp*AI site at position 1173, and *Bgl*II site at position 2730) in pCMV-P1 and -GTN. To generate the chimeric RNA1 construct containing Asn at position 253 and Ser at position 553 in the 1a protein, the region from *Sal*I to *Fsp*A1, was removed from pCMV-GTN-RNA1 by digesting with *Sal*I and *Fsp*AI and replaced with the corresponding fragment obtained from pCMV-P1-RNA1 by digesting with the same restriction enzymes. The resulting construct was named pCMV-RNA1-253N: 553S. To generate the chimeric RNA1 construct containing Asp at position 253 and Pro at position 553 in the 1a protein, the region from *Fsp*AI to *Bgl*II was removed from pCMV-GTN-RNA1 by digesting with *Fsp*AI and *Bgl*II and replaced with the corresponding fragment obtained from pCMV-P1-RNA1 by digesting with the same restriction enzymes. The resulting construct was named pCMV-RNA1-253D: 553P. Sequences of the chimeric constructs were validated by DNA sequencing.

### 2.4 Virus inoculation and detection

Infectious cDNA clones of CMV were inoculated by *Agrobacterium*-mediated infiltration (agroinfiltration). Briefrly, *Agrobacterium* transformants were grown at 28°C in LB medium containing 100 μg/ml kanamycin and 50 μg/ml rifampicin. *A. tumefaciens* cultures harboring either CMV RNA1, RNA2, or RNA3 were mixed in equal proportions for agroinfiltration as described previously (Seo et al. 2009a). The mixture was infiltrated into the abaxial surface of leaves using a 1-ml syringe. The inoculated plants were grown in an insect-free growth chamber. For mechanical inoculation of pepper, crude sap was prepared from systemic leaves of *N.benthamiana* infected with each CMV strain or isolate and rubbed on leaves dusted with carborundum (400 mesh). After inoculation, the leaves were washed with sterile water.

To verify systemic infection of CMV in the inoculated plants, total RNA was extracted from upper non-inoculated leaves and subjected to reverse-transcription polymerase chain reaction (RT-PCR) analysis using specific primers for CMV (5′-AAGAARCTTGTTTCGCGCATT-3′ and 5′-TGGTCTCCTTTTRAGGCCCCCA-3′) as described previously (Kim et al. 2014).

### 2.5 Sequence Analyses

Multiple sequence alignment was performed using ClustalW implemented in MEGA X software (Kumar et al., 2018). Determination of the best nucleotide substitution model was performed using the model selection program implemented in MEGA X. Pairwise genetic distance and diversity were analyzed using Tamura-Nei model in MEGA X. Phylogenetic analysis of CMV populations was performed using the maximum-likelihood method (Tamura-Nei model) in MEGA X with bootstrap values calculated using 1,000 random replications. Calculated trees were displayed using MEGA X. Median-joining haplotype networks were generated using NETWORK version 10 (https://www.fluxus-engineering.com/). Recombination events were analyzed using the RDP, GENECONV, Chimaera, MaxChi, BOOTSCAN, and SISCAN methods implemented in the RDP4 program with default settings and a Bonferroni corrected *P*-value cut-off of 0.01 as described previously (Seo et al. 2009c). To reduce the possibility of obtaining false recombination signals, only recombination events supported by at least three different methods with an associated *P*-value of <1.0 × 10^−6^ were considered.

### 2.6 Western blot analysis

Total protein was extracted from *N.benthamiana* leaves using TRIzol (Invitrogen) according to the manufacturer’s instructions. Proteins were separated by 12 per cent SDS-PAGE and transferred to a polyvinylidene difluoride (PVDF) membrane. The blot was probed with an antibody against CMV CP (Plant Virus Gene Bank, Korea). A secondary antibody, conjugated to horseradish peroxidase (Sigma-Aldrich, St. Louis, MO, USA), was used with the Amhersham ECL Western Blotting Detection System (GE Healthcare Life Sciences, Marlborough, MA, USA) to visualize the antigens.

## 3. Results

### 3.1 Biological and molecular characterization of newly emerged CMV variants that overcome resistance in pepper

CMV pathotypes P0 and P1 were prevalent in the 1980s–1990s and early 2000s, respectively, in Korean pepper fields and caused severe damage to the pepper industry (Choi et al. 2005; Lee et al. 2006). Since then, various pepper cultivars resistant to these pathotypes have been developed and cultivated in Korean fields, which has successfully and gradually decreased the incidence of CMV (Lee et al. 2009; Kang et al. 2012). However, since the early 2010s, CMV variants have emerged that can infect pepper cultivars resistant to CMV pathotype P1 (Kim et al. 2014; Choi et al. 2015).

In our previous study in 2013, we isolated and characterized the resistance-breaking CMV strain GTN from a pepper field in Goesan (Choi et al. 2015). Virulence analysis demonstrated that CMV-GTN could infect most commercial pepper cultivars (Choi et al. 2015). In 2016, we performed additional surveys in the same area to collect more resistance-breaking isolates. Six additional isolates were collected from pepper cultivars resistant to CMV pathotype P1 and their virulence was accessed on several pepper cultivars. CMV-Fny, -P1, and -GTN were included in the experiment as reference strains. All tested pepper cultivars, including Baerota and PR-Sagslee which are resistant to CMV pathotype P1, were susceptible to the six newly collected isolates (Table 1), indicating the new CMV variants were capable of overcoming resistance in pepper. Among them, three isolates (CMV-RB1, -RB2, and -RB3) were further analyzed to determine their complete genomic sequences. Genomic segment lengths were identical for all three isolates (3,358 nt for RNA1, 3,045 nt for RNA2, and 2,213 nt for RNA3). Their sequences were deposited in the GenBank database (accession numbers are listed in Table 2).

**Table 2.** CMV Korean isolates and reference strains analyzed in this study.

Country of origin	Collecting host	Strain or isolate	Sequencing year	Collection year	Accession number		
					RNA1	RNA2	RNA3
Korea	*Atractylodes macrocephala*	JC	2018	2018	MH594044	MH594045	MH594046
	*Canna generalis*	Can	2018	2018	LC381764	LC381763	LC381757
	*Capsicum annuum*	GTN	2016	2013	MN422336	MN422337	MN422338
		RB1	2017	2016	MT661448	MT661451	MT661454
		RB2	2017	2016	MT661449	MT661452	MT661455
		RB3	2017	2016	MT661450	MT661453	MT661456
		P1	2009	2004	MN422333	MN422334	MN422335
		RP1	2013	2007	KC527775	KC527685	KC527730
		RP3	2013	2007	KC527777	KC527687	KC527732
		RP4	2013	2007	KC527778	KC527688	KC527733
		RP5	2013	2007	KC527779	KC527689	KC527734
		RP7	2013	2007	KC527781	KC527691	KC527736
		RP8	2013	2007	KC527782	KC527692	KC527737
		RP9	2013	2007	KC527783	KC527693	KC527738
		RP14	2013	2007	KC527788	KC527698	KC527743
		RP15	2013	2007	KC527789	KC527699	KC527744
		RP16	2013	2007	KC527790	KC527700	KC527745
		RP18	2013	2007	KC527792	KC527702	KC527747
		RP22	2013	2007	KC527795	KC527705	KC527750
		RP23	2013	2007	KC527796	KC527706	KC527751
		RP25	2013	2007	KC527798	KC527708	KC527753
		RP26	2013	2007	KC527799	KC527709	KC527754
		RP27	2013	2007	KC527800	KC527710	KC527755
		RP28	2013	2007	KC527801	KC527711	KC527756
		RP38	2013	2007	KC527808	KC527718	KC527763
		RP44	2013	2007	KC527813	KC527723	KC527768
		RP47	2013	2007	KC527816	KC527726	KC527771
		RP48	2013	2007	KC527817	KC527727	KC527772
		RP49	2013	2007	KC527818	KC527728	KC527773
	*Cnidium officinale*	YY-Cnidium	2018	2018	LC424756	LC424757	LC424752
	*Cucurbita pepo*	Z1	2009	2004	GU327366	GU327367	GU327368
	*Glycine soja*	209	2014	2006	KJ400002	KJ400003	KJ400004
	*Ligusticum chuanxiong*	BH-Ligusticum	2019	2018	LC480453	LC480454	LC480455
	*Lilium longiflorum*	Li	2009	2009	AB506795	AB506796	AB506797
		LICB	2009	2009	AB506798	AB506799	AB506800
		Ly2	2002	1999	AJ535913	AJ535914	AJ296154
	*Melandryum firmum*	Mf	2000	1995	AJ276479	AJ276480	AJ276481
	*Passiflora edulis*	KoPF	2015	2012	KR535605	KR535606	KR535607
	*Rorippa palustris*	RPDJ	2015	2014	KT310080	KT310081	KT310082
	*Rudbeckia hirta*	Rb	2009	2005	GU327363	GU327364	GU327365
	*Solanum pseudocapsicum*	Sp	2018	2007	LC390165	LC3901606	LC390167
	*Stachys affinis*	Ack2	2019	2018	LC487907	LC487908	LC487909
	*Vigna angularis*	Va	2012	2004	JX014246	JX014247	JX014248
	*Zea mays*	ZM	2011	2006	JN180309	JN180310	JN180311
	*Zinnia elegans*	Ze	2018	2016	LC390004	LC390005	LC390006
USA	*Cucumis melo*	Fny	1990	1980	D00356	D00355	D10538
	*Lactuca saligna*	LS	2001	NA	AF416899	AF416900	AF127976
Australia	*Capsicum* sp.	Q	1985	1964	X02733	X00985	J02059
Philippines	*Ixora* spp.	Ix	1995	1972	U20220	U20218	U20219
China	*Brassica chinensis*	CTL	2007	NA	EF213023	EF213024	EF213025
Japan	*Nicotiana tabacum*	Y	1990	1954	D12537	D12538	M57602

### 3.2 Evolutionary analysis of resistance-breaking CMV variants in the CMV population

**Figure 1.**
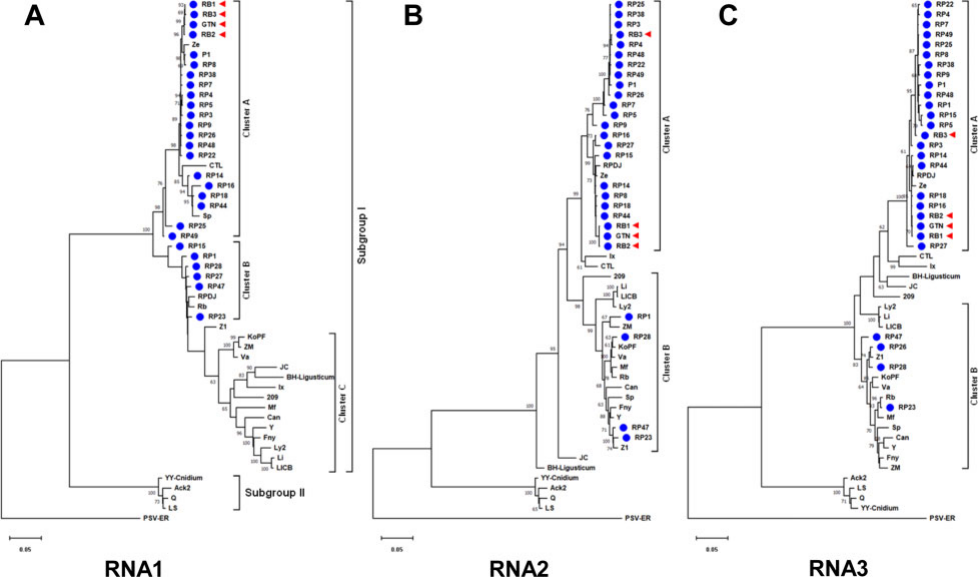
Phylogenetic analyses for the complete genome sequences of RNA1 (A), RNA2 (B), and RNA3 (C) of the Korean CMV population. Six CMV strains, CTL, Fny, Ix, Ls, Q, and Y, were included as reference strains. GenBank accession numbers of the analyzed CMV strains and isolates are available in Table 2. Peanut stunt virus strain ER (PSV-ER) was included as an out-group. GenBank accession numbers of PSV-ER: RNA1 (U15728), RNA2 (U15729), and RNA3 (U15730). Phylogenetic trees were reconstructed by the maximum-likelihood method applying the Tamura-Nei model method for nucleotide sequence analyses. Numbers on the branches indicate bootstrap percentages based on 1,000 replications (only values >70% are shown). CMV isolates collected from pepper are indicated with blue dots. Resistance-breaking variants of CMV emerged recently in pepper are indicated with red arrowheads.

To examine the evolutionary positions of emerging resistance-breaking CMV variants in the Korean CMV population, we performed phylogenetic analyses, including forty-five CMV strains and isolates collected from various host species in Korea. Regional-scale analysis of viral populations can provide more detailed aspects of evolution than those from a global-scale analysis. We also included six CMV strains (CTL, Fny, Ix, Ls, Q, and Y) as reference strains, and peanut stunt virus (PSV) strain ER as an outgroup taxon [GenBank accession numbers for PSV-ER: U15728 (RNA1), U15729 (RNA2), and U15730 (RNA3)]. Phylogenetic trees were reconstructed by the maximum-likelihood method based on alignments of the complete RNA1, RNA2, and RNA3 nucleotide sequences. All Korean CMV isolates except Ack2 and YY-Cnidium were found to belong to subgroup I (Fig. 1). When phylogenetic analyses were performed using RNA2 and RNA3 sequences, the Korean CMV isolates in subgroup I were largely subdivided into two clusters (Fig. 1B and C). In particular, most CMV isolates from pepper were grouped together in cluster A, while isolates from different hosts were grouped closely in cluster B (Fig. 1B and C). Divergence in the phylogenetic tree reconstructed with RNA1 sequences differed considerably from those reconstructed with RNA2 and RNA3 sequences (Fig. 1A). CMV isolates from pepper were further split into two clusters. CMV-GTN and all three resistance-breaking isolates, which were collected from pepper cultivars resistant to CMV pathotype P1, were closely grouped together in cluster A in the phylogeny of RNA1 (Fig. 1A). In contrast, CMV-RB3 was largely included in the cluster formed by pepper isolates in the phylogenies of RNA2 and RNA3, but not closely grouped with other resistance-breaking isolates and CMV-GTN (Fig. 1B and C).

**Figure 2.**
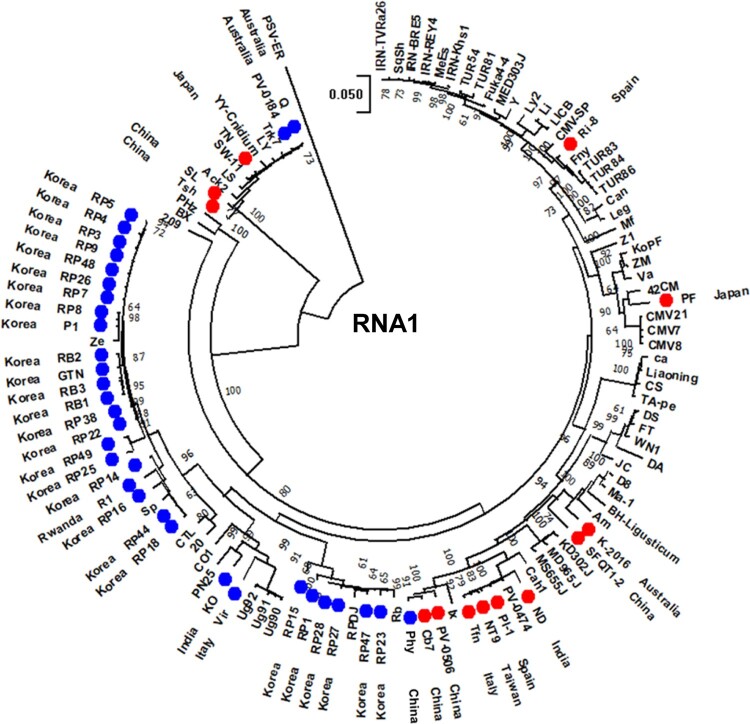
Phylogenetic analysis for the complete genome sequences of RNA1 of global CMV populations. GenBank accession numbers of the analyzed CMV strains and isolates are available in Supplementary Table S2. PSV-ER was included as an out-group. Phylogenetic trees were reconstructed by the maximum-likelihood method applying the Tamura-Nei model method for nucleotide sequence analyses. Numbers on the branches indicate bootstrap percentages based on 1,000 replications (only values >70% are shown). CMV isolates collected from pepper and tomato are indicated with blue and red dots, respectively. The country of origin is indicated next to the name of each CMV isolate.

The degree of divergence (i.e. branching and branch lengths) among isolates from different hosts was significantly higher in the RNA1 phylogenetic tree than those found in the RNA2 and RNA3 trees. Differences in divergence patterns were further supported by genetic diversity analysis (Table 3 and Supplementary Table S1), in which the Korean CMV isolates belonging to subgroup I were divided into two subpopulations based on their isolation hosts: pepper vs. other host plants. The results revealed higher genetic diversities between subpopulations (i.e. pepper vs. other) than within subpopulations (i.e. pepper vs. pepper) in all RNA segments (Table 3). In particular, the genetic diversity among CMV isolates from different hosts (i.e. other vs. other) and the mean diversity in entire population were significantly higher in RNA1 than in the other RNAs (Table 3 and Supplementary Table S1). This result suggests that different evolutionary constraints were applied on each CMV RNA, and RNA1 may have evolved under higher host-specific constraints than RNA2 and RNA3. Some patterns of host-associated phylogenetic relationships in CMV RNA1 were further evident when the CMV population, comprising 115 strains and isolates, was analyzed on a global scale (Fig. 2 and Supplementary Fig. S1). Most pepper isolates, including R1 (Rwanda), KO (India), and Vir (Italy), clustered together (Fig. 2). Another example can be found in the clustering of the CMV tomato isolates. Many tomato isolates clustered very closely together, even though their countries of origin varied (Fig. 2). Of note, CMV isolates 209 (from *Glycine soja*), BX (from *Pinellia ternate*), and PHz (from *P. ternate*) occupied intermediate positions between subgroups I and II (Fig. 2 and Supplementary Fig. S1), suggesting the existence of evolutionary intermediates between CMV subgroups I and II.

**Table 3.** Genetic diversity of the Korean CMV population.

Genome and subpopulation^a^	Nucleotide diversity^b^ within and between subpopulations	
	Pepper	Other
RNA1		
Pepper	0.045 ± 0.002	
Other	0.095 ± 0.004	0.094 ± 0.003
RNA2		
Pepper	0.047 ± 0.003	
Other	0.087 ± 0.004	0.072 ± 0.003
RNA3		
Pepper	0.030 ± 0.002	
Other	0.075 ± 0.004	0.064 ± 0.003

a CMV Korean isolates belonging to subgroup I were divided into two subpopulations on the basis of their isolation hosts: pepper vs. other host plants.

b Pairwise genetic diversity was analyzed by Tamura-Nei model using the MEGA X program. The numeric values indicate nucleotide diversity ± standard error.

**Figure 3.**
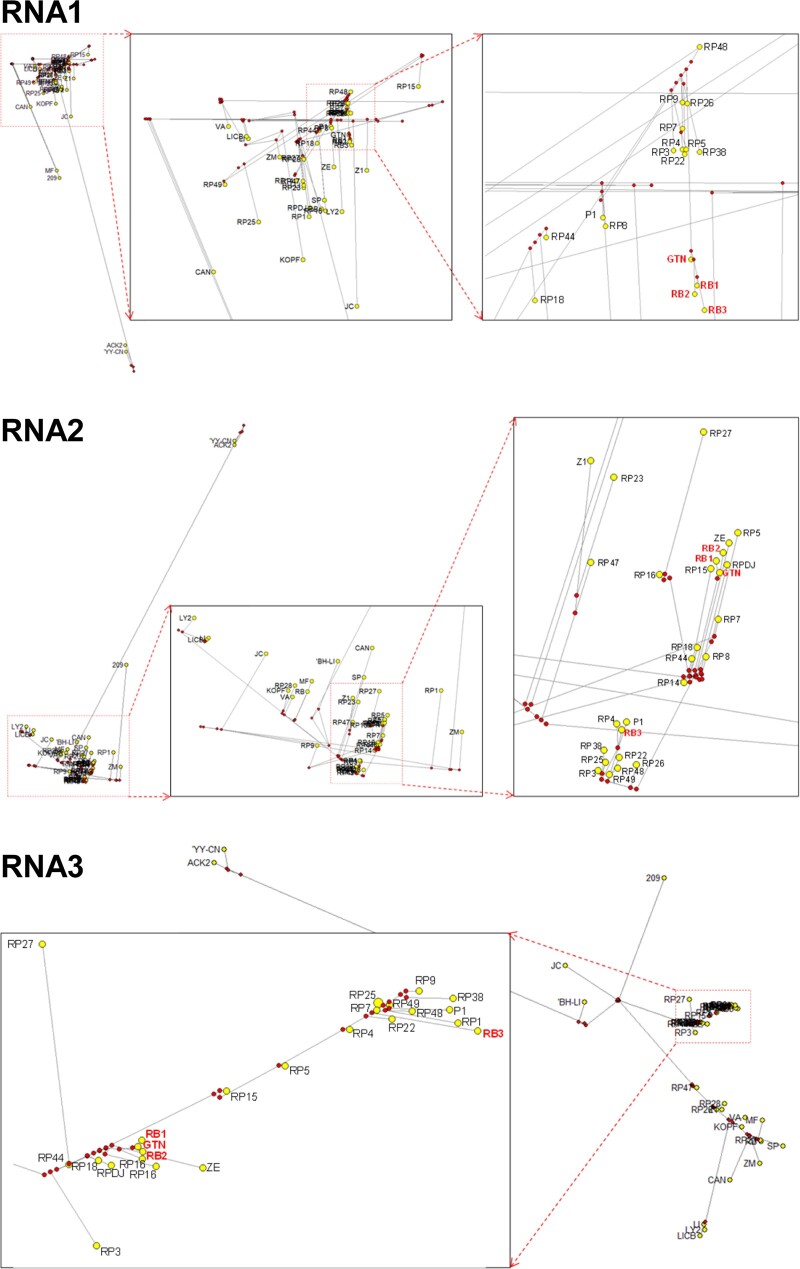
Median-joining haplotype network analyses for the complete genome sequences of RNA1, RNA2, and RNA3 of the Korean CMV population. Each yellow circle represents a haplotype, its size is proportional to haplotype frequency. Small red circles on nodes indicate median vectors that represent hypothetical missing or unsampled ancestral haplotypes.

Phylogenetic analyses using the maximum-likelihood method roughly showed that CMV resistance-breaking variants, including CMV-GTN, might be recently evolved from the existing CMV pepper population. Because no significant recombination events were detected in the resistance-breaking variants (data not shown), they might have emerged due to the marginal accumulation of mutations during replication. To obtain more insight into the ancestral relationships of resistance-breaking isolates in the Korean CMV population, median-joining haplotype network analyses were performed based on alignments of complete RNA1, RNA2, and RNA3 nucleotide sequences using the NETWORK software (Forster et al. 2020). The haplotype networks clearly showed that resistance-breaking isolates have originated from the pepper population very recently, as they clustered at or near the tips of the network (Fig. 3). CMV-GTN was likely the parental strain of CMV-RB1 and -RB2 in all RNA segments. For CMV-RB3, only RNA1 appeared to be derived from CMV-GTN, while RNA2 and RNA3 might have originated from different isolates by reassortment. More importantly, the resistance-breaking variants, including CMV-GTN, were of the same lineage and formed a clearly separated cluster in the RNA1 haplotype network (Fig. 3). This suggested that evolutionary selection in RNA1 might be in progress in the CMV pepper population and responsible for the emergence of resistance-breaking variants in Korea. In this regard, the recent increase in the cultivation of pepper cultivars resistant to CMV pathotypes P0 and P1 in Korea seems to be closely associated with evolutionary selection in the CMV pepper population.

### 3.3 Generation and pathogenicity characterization of an infectious cDNA clone of a resistance-breaking CMV variant

**Figure 4.**
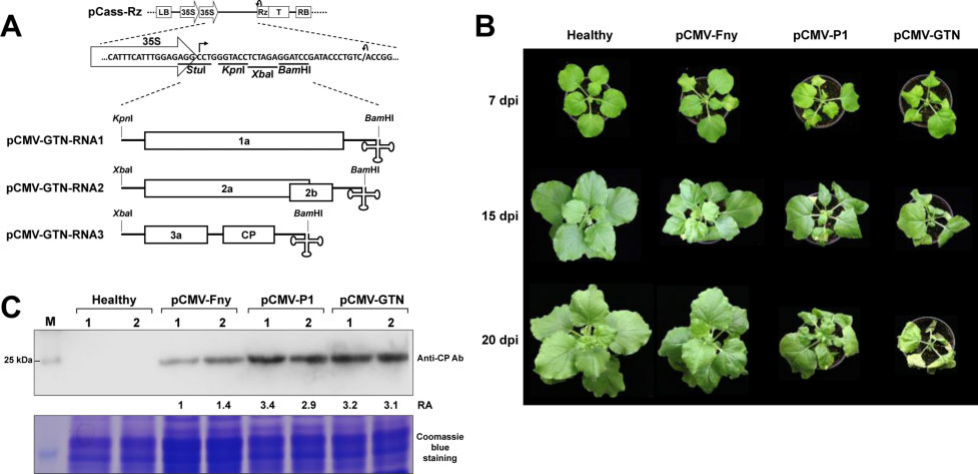
(A) Schematic representation of infectious cDNA clones of CMV-GTN. The pCassRz vector contains, in sequential order, a T-DNA left border (LB), a double 35S promoter, multiple cloning sites (*Stu*I, *Kpn*I, *Xba*I, and *Bam*HI), a *cis*-cleaving ribozyme sequence (Rz), a 35S terminator (T), and a T-DNA right border (RB). (B) Virulence of infectious cDNA clones of CMV-Fny, -P1, and -GTN in *Nicotiana benthamiana* observed at 7, 15, and 20 days post-infiltration (dpi). (C) Western blot analysis of CP accumulation of CMV. Total proteins were extracted from two individual *N.benthamiana* plants (numbers 1 and 2) infected with pCMV-Fny, -P1, or -GTN at 7 dpi and subjected to immunoblot analysis. Molecular weight marker (M) is indicated on the left. RA, relative accumulation levels of CP were calculated using ImageJ. Coomassie blue stained gel is shown below the blots as a loading control.

Infectious cDNA clones of two CMV strains F006Ey (pathotype P0) and P1 (pathotype P1), namely, pCMV-Fny and pCMV-P1, were obtained from our previous studies (Seo et al., 2009a; Kang et al. 2012). In this study, we additionally generated an infectious cDNA clone of CMV-GTN (pCMV-GTN), a parental resistance-breaking variant, to identify mutations responsible for resistance-breaking in pepper (Fig. 4A). We first sought to verify whether pCMV-GTN effectively infect susceptible hosts and has the same virulence as the original virus.

*N.benthamiana* plants were agro-infiltrated with pCMV-Fny, -P1, or -GTN and observed over a period of 4 weeks following the inoculation. At 7 days post-infiltration (dpi), *N.benthamiana* inoculated with pCMV-Fny exhibited mild mosaic symptoms in the upper systemic leaves, more severe symptoms of mosaic and leaf malformation were observed in the upper systemic leaves of *N.benthamiana* inoculated with pCMV-P1 and –GTN (Fig. 4B). Systemic infection with CMV-GTN was also confirmed by RT-PCR analysis using total RNA isolated from upper non-inoculated leaves (data not shown). These results demonstrated that pCMV-GTN was fully infectious upon agroinfiltration. Symptoms became more pronounced at 15 dpi, and symptomatic differences between the plants inoculated with pCMV-P1 and -GTN began to emerge (Fig. 4B). pCMV-GTN induced severe size reduction of the systemic leaves and necrosis on the inoculated leaves, while pCMV-P1 caused severe mosaic symptoms and malformation on the systemic leaves. At 20 dpi, the plants inoculated with pCMV-GTN exhibited necrosis on the leaves and petioles and growth was inhibited, whereas the plants inoculated with pCMV-P1 developed severely distorted systemic leaves but continued to grow (Fig. 4B). This result demonstrated the distinct pathogenicity of CMV-GTN compared to CMV-P1 and -Fny in *N.benthamiana*.

We next sought to examine whether the different pathogenicites among CMV strains were due to differences in their replication levels. To this end, western blot analysis was performed to compare CP accumulation levels using total proteins extracted from the symptomatic leaves of *N.benthamiana* plants infected with either pCMV-Fny, -P1, or -GTN at 7 dpi. CP accumulation levels of CMV-P1 and -GTN were much higher than that of CMV-Fny, but did not differ significantly between CMV-P1 and -GTN (Fig. 4C). This result suggests that differences in viral accumulation may be responsible for differences in pathogenicity between CMV-Fny and either CMV-P1 or -GTN. However, it is unlikely that pathogenicity differences between CMV-P1 and -GTN in *N.benthamiana* were due to viral accumulation.

We next examined the virulence of pCMV-GTN in various pepper cultivars. Crude sap from *N.benthamiana* infected with pCMV-Fny, -P1, or GTN was used as a viral source. Eleven commercial pepper cultivars were evaluated for resistance against each CMV strain. Symptom appearance in the inoculated plants were monitored for 4 weeks post-inoculation and RT-PCR was performed to confirm systemic viral infection. Two pepper cultivars, Baerota and PR-Sagslee, were resistant to both CMV-Fny and -P1, but susceptible to -GTN (Table 4). Five cultivars, Manita, Ogammanjok, Gilsang, Muhanjilju, and Superior, were only resistant to CMV-Fny. Four cultivars, Quarri, Sinhong, Chungyang, and Chungrok, were susceptible to all thee CMV strains, while none of the tested pepper cultivars were resistant to CMV-GTN (Table 4). The results confirmed that the infectious cDNA clones of CMV-GTN were fully infectious and demonstrated the same virulence as the original virus strain capable of infecting pepper cultivars resistant to CMV pathotypes P0 and P1.

**Table 4.** Virulence of infectious cDNA clones of CMV-GTN in various pepper cultivars.

CMV strain	Disease response of pepper cultivars to CMV inoculation^a^										
	Quarri	Sinhong	Chungyang	Chungrok	Manita	Ogammanjok	Gilsang	Muhanjilju	Superior	Baerota	PR-Sagslee
pCMV-Fny	S	S	S	S	R	R	R	R	R	R	R
pCMV-P1	S	S	S	S	S	S	S	S	S	R	R
pCMV-GTN	S	S	S	S	S	S	S	S	S	S	S

a S, susceptible (systemic mosaic and stunting); R, resistant; CMV infection was confirmed by RT-PCR analysis using total RNA isolated from upper non-inoculated leaves. Results were obtained from three independent experiments using at least three plants per experiment.

### 3.4 Identification of the genetic determinant of CMV responsible for resistance-breaking in pepper

**Figure 5.**
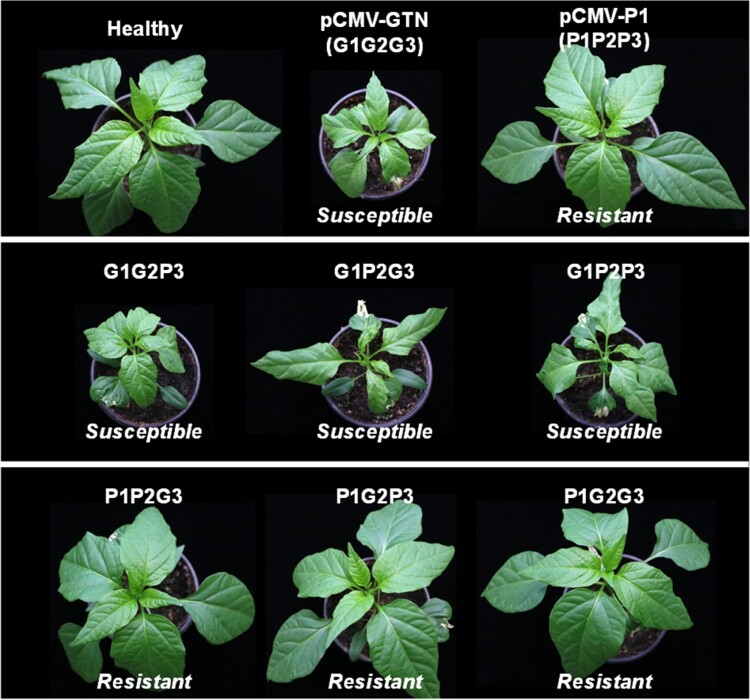
Virulence of pCMV-P1, -GTN, and their pseudo-recombinants in the pepper cultivar Baerota. G1, G2, and G3 represent pCMV-GTN RNA1, RNA2, and RNA3, while P1, P2, and P3 represent pCMV-P1 RNA1, RNA2, and RNA3, respectively. pCMV-GTN (G1G2G3) and three pseudo-recombinants (G1P2P3, G1G2P3, and G1P2G3) caused systemic infections in the pepper cultivar Baerota, whereas pCMV-P1 (P1P2P3) and three other pseudo-recombinants (P1G2G3, P1P2G3, and P1G2P3) were avirulent. Symptom development was monitored over 4 weeks post-inoculation and CMV infection was verified by RT-PCR using total RNA extracted from upper non-inoculated leaves.

The virulence of pCMV-GTN differs from that of pCMV-P1 in some pepper cultivars. Therefore, we examined which of the three genomic RNAs was the genetic determinant causing resistance breaking. To this end, the virulence of pseudo-recombinants between pCMV-GTN and -P1 was examined in pepper plants by mixing *Agrobacterium* cultures harboring plasmids expressing each RNA segment of CMV-GTN and CMV-P1: G1, G2, and G3 represent pCMV-GTN RNA1, RNA2, and RNA3, while P1, P2, and P3 represent pCMV-P1 RNA1, RNA2, and RNA3, respectively. Virulence of six pseudo-recombinants (G1G2P3, G1P2G3, G1P2P3, P1G2G3, P1G2P3, and P1P2G3) was evaluated in four pepper cultivars, including Quarri, Sinhong, Manita, and Baerota. Symptom development was monitored over 4 weeks post-inoculation and CMV infection was confirmed by RT-PCR using total RNA extracted from upper non-inoculated leaves. All pseudo-recombinants caused systemic infection in pepper cultivars Quarri, Sinhong, and Manita, which are susceptible to both pCMV-GTN and -P1 (Table 5). On the other hand, three pseudo-recombinants (G1P2P3, G1G2P3, and G1P2G3) containing pCMV-GTN RNA1 caused systemic infections in the pepper cultivar Baerota, which is resistant to CMV-P1 but susceptible to CMV-GTN (Table 5 and Fig. 5). In comparison, three other pseudo-recombinants (P1G2G3, P1P2G3, and P1G2P3) were avirulent in this pepper cultivar (Table 5 and Fig. 5). Briefly, the results indicate that RNA1 of CMV-GTN contained the mutations responsible for breaking resistance in pepper.

**Table 5.** Virulence of pseudo-recombinants between CMV-GTN and -P1 in various pepper cultivars.

Inoculum^a^	Disease response of pepper cultivars to CMV inoculation^b^			
	Quarri	Sinhong	Manita	Baerota
G1G2G3	S	S	S	S
G1G2P3	S	S	S	S
G1P2G3	S	S	S	S
G1P2P3	S	S	S	S
P1G2G3	S	S	S	R
P1G2P3	S	S	S	R
P1P2G3	S	S	S	R
P1P2P3	S	S	S	R

a G1, G2, and G3 represent pCMV-GTN RNA1, RNA2, and RNA3, while P1, P2, and P3 represent pCMV-P1 RNA1, RNA2, and RNA3, respectively.

b S, susceptible (systemic mosaic and stunting); R, resistant; CMV infection was confirmed by RT-PCR analysis using total RNA isolated from upper non-inoculated leaves. Results were obtained from three independent experiments using at least three plants per experiment.

**Figure 6.**
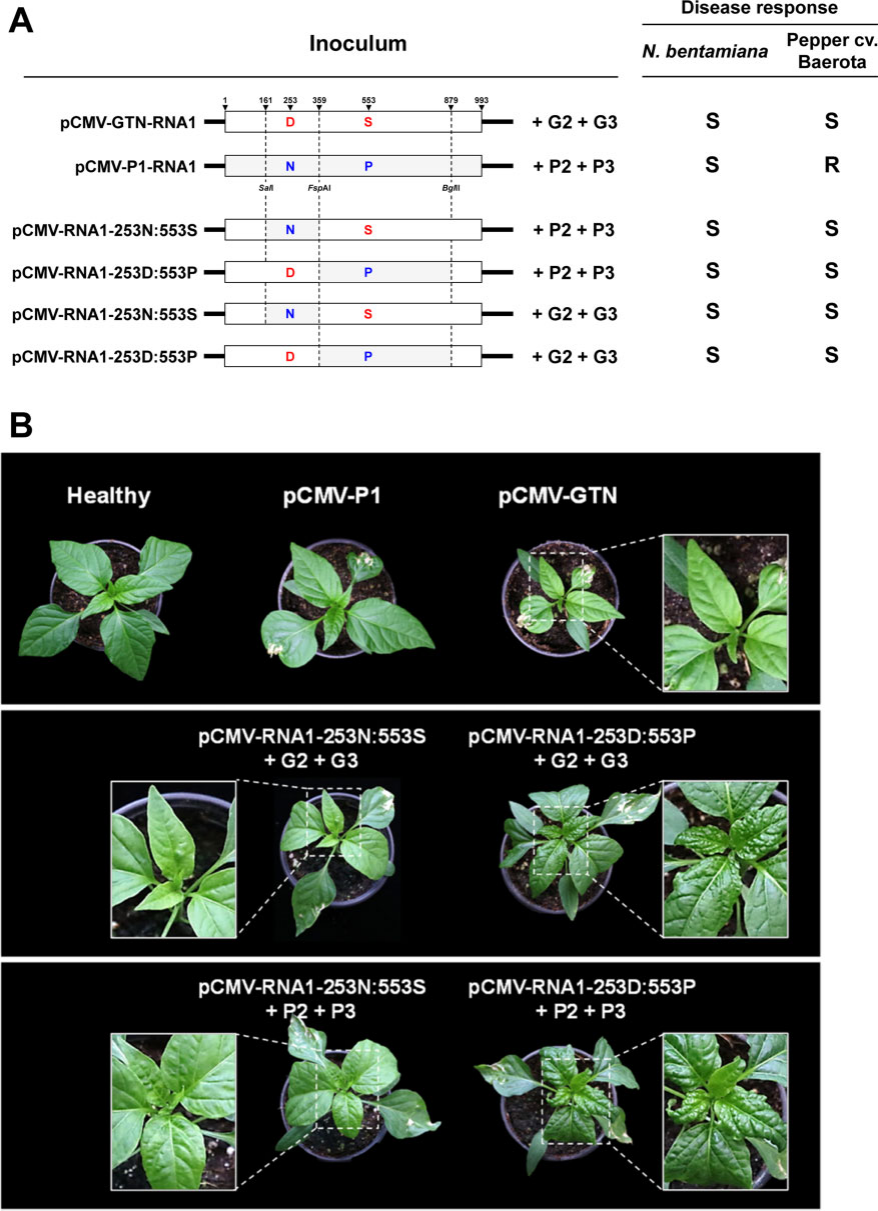
Schematic representation of chimeric RNA1 constructs between pCMV-GTN and -P1 of CMV and their virulence in the pepper cultivar Baerota. Two chimeric RNA1 contructs between CMV-GTN and -P1 were generated by exchanging corresponding fragments. The positions of the restriction enzyme cleavage sites used to make the chimeric constructs are shown. pCMV-GTN- and pCMV-P1-derived regions are indicated by white and gray boxes, respectively. pCMV-RNA1-253N:553S contains Asn at position 253 and Ser at position 553 in the 1a protein. pCMV-RNA1-253D:553P contains Asp at position 253 and Pro at position 553 in the 1a protein. Each chimeric RNA1 mutant was inoculated in combination with either pCMV-GTN-RNA2 (G2) and -RNA3 (G3) or pCMV-P1-RNA2 (P2) and -RNA3 (P3). Symptom development was monitored over 4 weeks post-inoculation and CMV infection was verified by RT-PCR using total RNA extracted from upper non-inoculated leaves. S, susceptible (systemic infection); R, resistant.

Since CMV RNA1 encodes only the 1a protein, we hypothesized that amino acid residue differences between the 1a proteins of CMV-GTN and -P1 might be responsible for the observed virulence differences. Thus, the amino acid sequences of the 1a proteins of the two strains were compared. Sequence analysis revealed only two amino acids differed between the 1a proteins for CMV-GTN and -P1 (at amino acid positions 253 and 553) (Fig. 6A and Supplementary Fig. S2). Interestingly, these amino acid substitutions were caused by nonsynonymous mutations at nucleotide positions 852 and 1752 in RNA1, respectively, and the same mutations were also found in other resistance-breaking isolates examined in this study (Supplementary Fig. S3). To identify which amino acid difference in the 1a protein affected virulence, single amino acid substitution mutants were generated by exchanging corresponding RNA1 genomic regions between pCMV-GTN and -P1, utilizing commonly available restriction enzyme sites (Fig. 6A). Virulence of these mutants was evaluated in two pepper cultivars, Quarri and Baerota. Symptom development was monitored for 4 weeks post-inoculation. As with their parental viruses, pCMV-RNA1-253N: 553S and -253 D: 553P were virulent in *N.benthamiana* when inoculated in combination with either P2 + P3 or G2 + G3 (Fig. 6A). In addition, both mutants infected Baerota systemically, demonstrating single amino acid substitutions at amino acid positions 253 and 553 independently affected CMV pathogenicity in pepper (Fig. 6A and B). Interestingly, regardless of the combination with RNA2 and RNA3, pCMV-RNA1-253N: 553S induced symptoms similar to those induced by pCMV-GTN in Baerota, whereas pCMV-RNA1-253D: 553P caused distinct symptoms of embossed mosaic on young leaves (Fig. 6B). Thus, it appears that the amino acid residue at position 553 of the 1a protein was associated with symptomatic variation of CMV in pepper. Maintenance of the amino acid substitutions of the 1a protein in the progeny viruses was confirmed by RT-PCR amplification of the corresponding genomic regions, followed by direct sequencing.

## 4. Discussion

Mutation pressure, natural selection, and genetic drift are the main evolutionary forces responsible for adaptive population diversity (Gao et al. 2017). The population of RNA viruses is diverse due to their error-prone replication and short generation times (Roossinck 2003). Thus, selective pressures exerted by various ecological and genetic factors on virus–host interactions play a critical role in shaping the virus population structure and may lead to virulence evolution. These evolutionary consequences strongly influence viral fitness and virulence in a particular host genotype and can result in host adaptation (Rico et al. 2006; Safari and Roossinck 2018).

CMV is distributed worldwide and has the most extensive host range of any plant virus, infecting more than 1,200 plant species, including over 200 types of mono- and dicotyledonous crop plants (Palukaitis and Garcia-Arenal 2003). This suggests that CMV may have the great evolutionary capacity, allowing it to adapt to new hosts and environments rapidly. Our phylogenetic analyses of the Korean CMV population revealed genetic evidence for the host-adaptive evolution of CMV (Fig. 1). In particular, most CMV isolates from pepper were genetically grouped together and their degree of divergence was significantly low, suggesting that host-driven selective constraints may restrict CMV population diversity in pepper (Fig. 1 and Table 3). Further, our global-scale phylogenetic analysis of the CMV population structure revealed some patterns of host-adaptive evolution of CMV in pepper and tomato (Fig. 2 and Supplementary Fig. S1). The host-adaptive evolution of CMV was previously suggested after the biological characterization of some CMV isolates from lily and soybean, as these isolates demonstrated restricted host range (Masuta et al. 2002; Hong et al. 2007; Lee et al. 2007). Many CMV strains induced mosaic symptoms in tobacco plants and systemically infected cucurbits but not lilies; however, CMV lily isolates (HL, Ly2, and Ly8) and were unable to infect cucurbits and displayed distinct pathogenicity in tobacco plants (Masuta et al. 2002; Lee et al. 2007). Host adaptation of CMV lily isolates was evident at the genetic level since they formed a separated cluster within subgroup I in the phylogenetic trees (Figs 1 and 2). Similarly, some CMV isolates from soybean demonstrated specific host range and distinct pathogenicity (Hong et al. 2007). Phylogenetic analyses suggested that the MP of CMV soybean isolates was under high evolutionary constraint (Hong et al. 2007). Since the MP is involved in virus movement within a host, host-imposed constraints might affect the evolution of the MP gene.

The divergence level in RNA1 was significantly higher than in other RNAs in the Korean CMV population (Table 3), which suggests that each CMV RNA has evolved under different constraints. Each viral component being subjected to different evolutionary dynamics has been reported for various RNA viruses (Garcia-Arenal et al. 2001; Seo et al. 2009c; Kim et al. 2014). Viruses are obligate parasites that require numerous host factors at every stage of infection; therefore, the degree of selective constraint on a viral protein is often highly associated with its interactions with host factors. CMV RNA1 encodes the 1a protein, which is entirely associated with vacuolar membranes, but contains no transmembrane domains, indicating that the 1a protein interacts with host proteins on vacuolar membranes to be recruited (Palukaitis and Garcia-Arenal 2003). Thus, it seems likely that evolution of the 1a protein is highly constrained by compatibility with host-interacting proteins. Our phylogenetic and diversity analyses of the Korean CMV population suggest that host-imposed constraints on RNA1 have likely played an essential role in the host-adaptive evolution of CMV and in shaping the structure of the CMV population.

In crop fields, genotypic variations in host plant resistance can significantly influence virus population dynamics and evolution, because host resistance can act as a selective pressure on the virus population, forcing rapid adaptation to new circumstances (Fabre et al. 2009; Janzac et al. 2009; Jones 2009). Indeed, the widespread use of resistant cultivars has contributed to the emergence of resistance-breaking variants of several plant viruses (Harrison 2002; Garcia-Arenal and McDonald 2003; Elena et al. 2014). Our phylogenetic analyses revealed that CMV resistance-breaking variants might have recently evolved from the CMV pepper population (Fig. 1). In Korea, the deployment of host resistance has been applied to control CMV in pepper fields since the 1990s (Choi et al. 2005; Lee et al. 2009). However, resistance-breaking variants have continuously emerged and CMV continues to cause the most severe damage in Korean pepper crops (Lee et al. 2006; Kim et al. 2014; Choi et al. 2015). Our haplotype network analyses suggested that selective evolution in RNA1 appears to be in progress in the CMV pepper population (Fig. 3). In Korea, breeding and cultivation of pepper cultivars resistant to CMV pathotypes P0 and P1 have gradually increased since the identification of the dominant genes that confer resistance to these CMV pathotypes in the early 2010s (Choi et al. 2018; Kang et al. 2010). Therefore, the recent emergence of CMV resistance-breaking variants and selective evolution in RNA1 may be a result of deployed resistance as a selective pressure in pepper fields. Although most mutations are negatively selected in susceptible hosts, a lineage carrying beneficial mutations can arise when host resistance exerts strong selective pressures on a virus population to overcome that resistance, resulting in resistance-breaking (Elena et al. 2014).

To identify mutations responsible for resistance-breaking in pepper, we utilized infectious cDNA clones of two representative CMV strains with different virulences in pepper. By analyzing the virulence of pseudo-recombinants and chimeric mutants between the two strains, we demonstrated that two nonsynonymous mutations in RNA1 (at nucleotide positions 852 and 1752) are responsible for resistance-breaking in pepper (Fig. 6 and Supplementary Fig. S3). These nonsynonymous mutations caused amino acid substitutions at positions 253 and 553 in the 1a protein encoded in RNA1, respectively. Therefore, this result proved our assumption obtained from genetic analyses of the CMV population for the mechanism underlying the recent emergence of resistance-breaking variants in pepper. In other words, the high-divergence capacity of RNA1 may enhance the host-adaptive evolution of CMV and the rapid appearance of resistance-breaking variants. Alterations in CMV virulence upon nonsynonymous mutations in RNA1 have been shown in previous studies (Diveki et al. 2004; Kang et al. 2012; Tian et al. 2020). Amino acid substitutions at the C-terminus of the 1a protein were responsible for overcoming *Cmr1*-based resistance by CMV-P1 in pepper (Kang et al. 2012). In addition, several single amino acid substitutions in the 1a protein were independently responsible for determining the pathogenicity of CMV in tobacco and *Arabidopsis* (Diveki et al. 2004; Tian et al. 2020). Although the majority of nonsynonymous mutations in viral genes are deleterious because they can negatively affect normal gene function at the protein level, some rare nonsynonymous mutations with neutral or minimal effects may result in overcoming host resistance by abolishing interactions with resistance proteins (Elena et al. 2014; Moreno-Perez et al. 2016). Nonsynonymous mutations at positions 852 and 1752 in RNA1 demonstrated no significant effects on the normal function of the 1a protein because both CMV-P1 and -GTN successfully infected susceptible host plants (Fig. 6). Instead, these mutations are more likely to abolish recognition of the 1a protein by unidentified resistance proteins, thereby causing resistance breaking. In addition, while amino acid mutations at positions 253 and 553 independently conferred virulence to CMV in the pepper cultivar Baerota (Fig. 6B), each mutation affected the pathogenicity of CMV differently as demonstrated by distinct symptoms induced by CMV mutants carrying each mutation. In particular, the amino acid substitution from Pro to Ser at position 553 in the 1a protein resulted in changing symptomatology as well as virulence (Fig. 6B). Alteration of virus pathogenicity by a single amino acid change has been observed in many viruses (Lewandowski and Dawson 1993; Shintaku et al. 1992; Masuta et al. 1999; Seo et al. 2009b). In CMV, alteration in symptom severity was also observed when the single amino acid substitution of Pro for Ser at position 129 was introduced into the CP (Shintaku 1991). Because Pro and Ser frequently substitute for each other in nature, they may have similar conformational characteristics in protein structures (Petsko and Ringe 2004; Schroter et al. 2014). As substitutions from Pro to Ser in the 1a protein demonstrated no significant effect on the infectivity of CMV in the susceptible host, *N.benthamiana* (Fig. 6), it is likely that the amino acid at position 129 in the 1a protein may be involved in interactions with host partners that are associated with pathogenicity in pepper.

Understanding the mechanisms that influence the evolutionary direction of virus populations is essential for the development of more durable strategies to control viral diseases in crop fields. In pepper, various sources of resistance to CMV have been identified and utilized to breed resistant cultivars (Caranta et al. 1997; Caranta et al. 2002; Kang et al. 2010; Yao et al. 2013; Choi et al. 2018). Despite extensive efforts to control CMV by deploying resistance in the field, variants capable of overcoming deployed resistance have continuously emerged, and CMV remains the prevalent and most destructive virus in pepper crops. Resistance-driven selective pressure combined with the high evolutionary capacity of CMV might have contributed to the unique evolution of CMV in pepper. Our molecular genetic analyses of resistance-breaking CMV variants suggest that the evolution of the CMV population driven by host resistance is ongoing in the pepper field. Our results also suggest that overreliance on the deployment of a single resistance gene may reduce resistance durability against CMV in the short term. Therefore, more integrated approaches that complement host resistance are necessary for successful control of CMV in pepper.

## Data availability

All data are available within this manuscript and its supplementary materials.

## Supplementary Data

Supplementary data are available at *Virus Evolution* online.

## Supplementary Material

veaa070_Supplementary_Data
