# Supplementary material for: Newly emerged resistance-breaking variants of cucumber mosaic virus represent ongoing host-interactive evolution of an RNA virus
Source: Virus Evol. 2020 Nov 7;6(2):veaa070. doi: 10.1093/ve/veaa070 (PMC7673075; doi:10.1093/ve/veaa070)
Supplement: veaa070_Supplementary_Data [file veaa070_supplementary_data.zip › Supplementary Tables.docx]

Supplementary Table S1. Genetic diversity of the Korean CMV population.

| Genome and subpopulation^a^ | Mean diversity^b^ in entire population | Mean interpopulation diversity | Coefficient of differentiation | Mean diversity within subpopulations |
| --- | --- | --- | --- | --- |
| R1 | 0.076 ± 0.003 | 0.006 ± 0.001 | 0.079 ± 0.008 | 0.070 ± 0.002 |
| R2 | 0.069 ± 0.003 | 0.010 ± 0.001 | 0.141 ± 0.008 | 0.060 ± 0.002 |
| R3 | 0.056 ± 0.003 | 0.009 ± 0.001 | 0.165 ± 0.013 | 0.047 ± 0.002 |

^a^ CMV Korean isolates belonging to subgroup I were divided into two subpopulations on the basis of their isolation hosts: pepper vs. other host plants.

^b^ Pairwise genetic diversity was analyzed by Tamura-Nei model using the MEGA X program. The numeric values indicate nucleotide diversity ± standard error.

Supplementary Table S2. Global CMV strains and isolates analyzed in this study.

| Country of origin | Collecting host | | | Strain or isolate | | Sequencing year | | Collection year | | Accession number | | | | | |
| --- | --- | --- | --- | --- | --- | --- | --- | --- | --- | --- | --- | --- | --- | --- | --- |
|  |  |  |  |  |  |  |  |  |  | RNA1 | | RNA2 | | RNA3 | |
| Australia | *Capsicum sp.* | | | PV-0184 | | 2016 | | NA | | KX525730 | | KX525734 | | KX525738 | |
|  |  |  |  | Q | | 1985 | | 1964 | | X02733 | | X00985 | | J02059 | |
|  | *Cymbidium sp.* | | | SW-11 | | 2014 | | 2012 | | KM434204 | | KM434205 | | KM434206 | |
|  | *Lupinus angustifolius* | | | LY | | 1999 | | NA | | AF198101 | | AF198102 | | AF198103 | |
|  | *Solanum lycopersicum* | | | K-2016 | | 2017 | | 2016 | | MG182148 | | MG182149 | | MG182150 | |
| Brazil | *Spinach oleracea* | | | CMV-SP | | 2017 | | 2014 | | KY886409 | | KY886410 | | KY886411 | |
| China | *Arachis hypogaea* | | | ca | | 2003 | | NA | | AY429434 | | AY429433 | | AY429432 | |
|  |  |  |  | CS | | 2003 | | NA | | AY429435 | | AY429436 | | AY429437 | |
|  |  |  |  | Liaoning | | 2018 | | 2017 | | MH394188 | | MH394189 | | MH394190 | |
|  |  |  |  | TA-pe | | 2019 | | 2017 | | MK415656 | | MK415657 | | MK415658 | |
|  | *Atractylodes macrocephala* | | | Am | | 2012 | | 2011 | | JX993909 | | JX993910 | | JX993911 | |
|  | *Brassica chinensis* | | | CTL | | 2007 | | NA | | EF213023 | | EF213024 | | EF213025 | |
|  | *Canna sp.* | | | Cah1 | | 2008 | | 2007 | | FJ268744 | | FJ268745 | | FJ268746 | |
|  | *Capsicum frutescens* | | | Phy | | 2006 | | NA | | DQ402477 | | DQ412731 | | DQ412732 | |
|  | *Pinellia ternate* | | | BX | | 2006 | | NA | | DQ399548 | | DQ399549 | | DQ399550 | |
|  |  |  |  | PHz | | 2008 | | 2007 | | EU723568 | | EU723570 | | EU723569 | |
|  | *Piper nigrum* | | | DA | | 2015 | | NA | | KU255790 | | KU255791 | | KU255792 | |
|  |  |  |  | DS | | 2015 | | NA | | KU255787 | | KU255788 | | KU255789 | |
|  |  |  |  | FT | | 2015 | | NA | | KU255784 | | KU255785 | | KU255786 | |
|  |  |  |  | WN1 | | 2015 | | NA | | KT004542 | | KT004543 | | KT004544 | |
|  | *Solanum lycopersicum* | | | Cb7 | | 2007 | | NA | | EF216866 | | DQ785470 | | EF216867 | |
|  |  |  |  | PV-0506 | | 2016 | | NA | | KX525728 | | KX525732 | | KX525736 | |
|  |  |  |  | SFQT1-2 | | 2010 | | NA | | HQ283392 | | HQ283391 | | HQ283393 | |
|  |  |  |  | SL | | 2016 | | 2014 | | KX013370 | | KX013371 | | KX013372 | |
|  |  |  |  | Tsh | | 2006 | | 2005 | | EF202595 | | EF202596 | | EF202597 | |
| Hungary | *Trifolium repens* | | | Trk7 | | 1998 | | 1978 | | AJ007933 | | AJ007934 | | L15336 | |
| India | *Capsicum annuum* | | | KO | | 2014 | | 2013 | | KM272277 | | KM272278 | | KM272275 | |
|  | *Plectranthus amboinicus* | | | CO1 | | 2019 | | 2018 | | MK482376 | | MK482377 | | MK482378 | |
|  | *Piper nigrum* | | | PN25 | | 2016 | | 2003 | | KU947029 | | KU947030 | | KU947031 | |
|  | *Solanum lycopersicum* | | | ND | | 2009 | | NA | | GU111227 | | GU111228 | | GU111229 | |
| Iran | *Cucumis melo* | | | MeEs | | 2018 | | 2017 | | MH782235 | | MH782236 | | MH782237 | |
|  | *Cucurbita pepo* | | | SqSh | | 2018 | | 2017 | | MH782238 | | MH782239 | | MH782240 | |
|  | *Raphanus sativus* | | | IRN-BRE5 | | 2015 | | 2007 | | LC066459 | | LC066460 | | LC066461 | |
|  |  |  |  | IRN-Khs1 | | 2015 | | 2004 | | LC066462 | | LC066463 | | LC066464 | |
|  |  |  |  | IRN-REY4 | | 2015 | | 2004 | | LC066465 | | LC066466 | | LC066467 | |
|  | *Rapistrum rugosum* | | | IRN-TVRa26 | | 2015 | | 2006 | | LC066480 | | LC066481 | | LC066482 | |
| Italy | | *Capsicum sp.* | Vir | | 2012 | | NA | | HE962478 | | HE962479 | | HE962480 | |  |
|  |  | *Solanum lycopersicum* | Tfn | | 1998 | | NA | | Y16924 | | Y16925 | | Y16926 | |  |
| Japan | | *Cucumis sativus* | 42CM | | 2007 | | 1986 | | AB368496 | | AB368497 | | AB368498 | |  |
|  |  |  | Fuka4-4 | | 2004 | | NA | | AB188231 | | AB188232 | | AB188233 | |  |
|  |  | *Nicotiana tabacum* | Y | | 1990 | | 1954 | | D12537 | | D12538 | | M57602 | |  |
|  |  | *Raphanus sativus* | D8 | | 2004 | | NA | | AB179764 | | AB179765 | | AB004781 | |  |
|  |  |  | KD302J | | 2015 | | 2006 | | LC066411 | | LC066412 | | LC066413 | |  |
|  |  |  | MD965J | | 2015 | | 2001 | | LC066414 | | LC066415 | | LC066416 | |  |
|  |  |  | MED303J | | 2015 | | 2001 | | LC066417 | | LC066418 | | LC066419 | |  |
|  |  | *Spinach oleracea* | MS655J | | 2015 | | 2007 | | LC066420 | | LC066421 | | LC066422 | |  |
|  |  | *Solanum lycopersicum* | PF | | 2007 | | 1982 | | AB368499 | | AB368500 | | AB368501 | |  |
|  |  |  | TN | | 2004 | | NA | | AB176849 | | AB176848 | | AB176847 | |  |
|  |  | *Vigna unguiculata* | Leg | | 1993 | | NA | | D16403 | | D16406 | | D16405 | |  |
|  |  | *Zea mays* | Ma-1 | | 2017 | | NA | | LC339509 | | LC339510 | | LC339511 | |  |
| Philippines | | *Ixora spp.* | Ix | | 1995 | | 1972 | | U20220 | | U20218 | | U20219 | |  |
| Poland | | *Cucurbita pepo* | CMV7 | | 2018 | | NA | | MG882747 | | MG882748 | | MG882749 | |  |
|  |  |  | CMV8 | | 2018 | | NA | | MG882750 | | MG882751 | | MG882752 | |  |
|  |  |  | CMV21 | | 2018 | | NA | | MG882753 | | MG882754 | | MG882755 | |  |
| Rwanda | | *Capsicum sp.* | R1 | | 2017 | | 2016 | | MG470798 | | MG470799 | | MG470800 | |  |
| Spain | | *Cucumis melo* | PV-0474 | | 2016 | | NA | | KX525729 | | KX525733 | | KX525737 | |  |
|  |  | *Solanum lycopersicum* | Pl-1 | | 2006 | | NA | | AM183114 | | AM183115 | | AM183116 | |  |
|  |  |  | Ri-8 | | 2006 | | NA | | AM183117 | | AM183118 | | AM183119 | |  |
| Taiwan | | *Musa sp.* | 20 | | 2018 | | 2007 | | MH709142 | | MH709143 | | MH709144 | |  |
|  |  | *Solanum lycopersicum* | NT9 | | 1994 | | 1988 | | D28778 | | D28779 | | D28780 | |  |
| Turkey | | *Raphanus sativus* | TUR54 | | 2015 | | 2007 | | LC066501 | | LC066502 | | LC066503 | |  |
|  |  |  | TUR81 | | 2015 | | 2007 | | LC066504 | | LC066505 | | LC066506 | |  |
|  |  | *Rapistrum rugosum* | TUR83 | | 2015 | | 2007 | | LC066507 | | LC066508 | | LC066509 | |  |
|  |  |  | TUR84 | | 2015 | | 2007 | | LC066510 | | LC066511 | | LC066512 | |  |
|  |  |  | TUR86 | | 2015 | | 2007 | | LC066513 | | LC066514 | | LC066515 | |  |
| Uganda | | *Xanthosoma sp.* | Ug90 | | 2017 | | 2015 | | MG021454 | | MG021455 | | MG021456 | |  |
|  |  |  | Ug91 | | 2017 | | 2015 | | MG021457 | | MG021458 | | MG021459 | |  |
|  |  |  | Ug92 | | 2017 | | 2015 | | MG021460 | | MG021461 | | MG021462 | |  |
| USA | | *Cucumis melo* | Fny | | 1990 | | 1980 | | D00356 | | D00355 | | D10538 | |  |
|  |  | *Lactuca saligna* | LS | | 2001 | | NA | | AF416899 | | AF416900 | | AF127976 | |  |
