# Supplementary material for: Newly emerged resistance-breaking variants of cucumber mosaic virus represent ongoing host-interactive evolution of an RNA virus
Source: Virus Evol. 2020 Nov 7;6(2):veaa070. doi: 10.1093/ve/veaa070 (PMC7673075; doi:10.1093/ve/veaa070)
Supplement: veaa070_Supplementary_Data [file veaa070_supplementary_data.zip › Supplementary_Figures.pdf]

Supplementary Figure S1

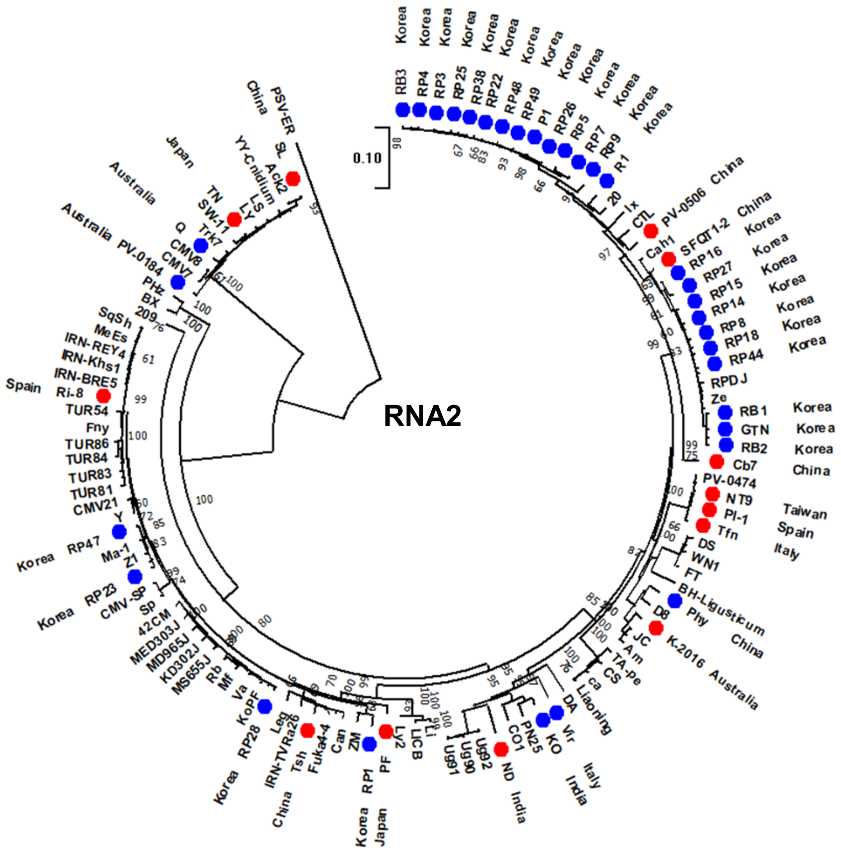

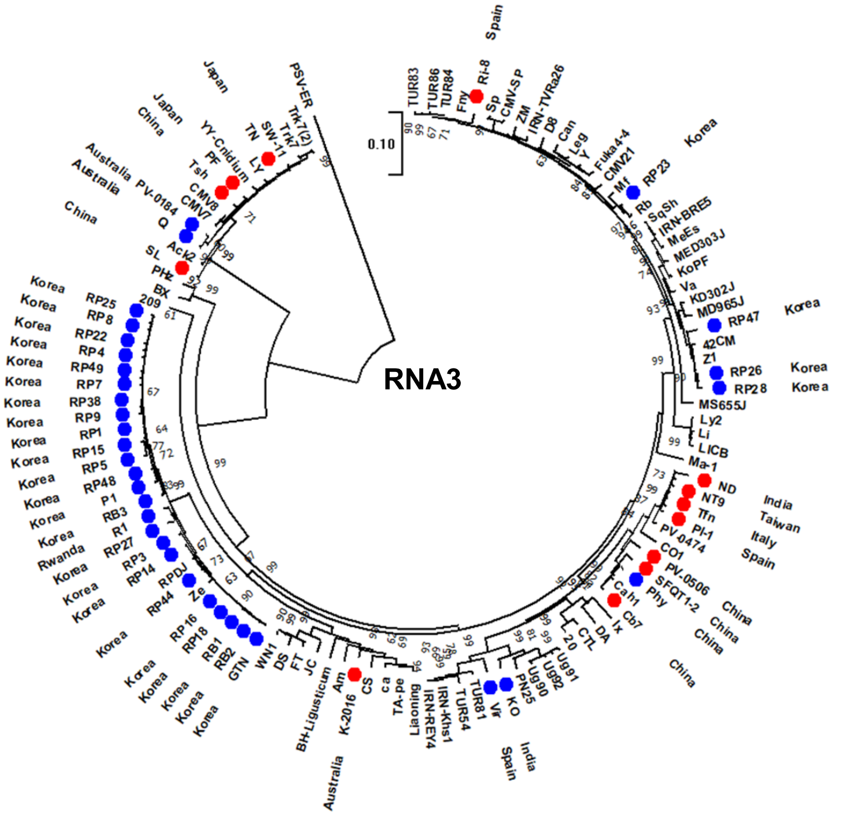

Supplementary Figure S1. Phylogenetic analyses for complete genome sequences of RNA2 and RNA3 for global CMV populations. GenBank accession numbers of the analyzed CMV strains and isolates are available in Supplementary Table S1. Peanut stunt virus strain ER (PSV-ER) was included as an out-group. GenBank accession numbers of PSV-ER: RNA1 (U15728), RNA2 (U15729), and RNA3 (U15730). Phylogenetic trees were reconstructed by the maximum-likelihood method applying the Tamura-Nei model method for nucleotide sequence analyses. Numbers on the branches indicate bootstrap percentages based on 1,000 replications (only values >70% are shown). CMV isolates collected from pepper and tomato are indicated with blue and red dots, respectively. The country of origin is indicated next to the name of each CMV isolate.

Supplementary Figure S2

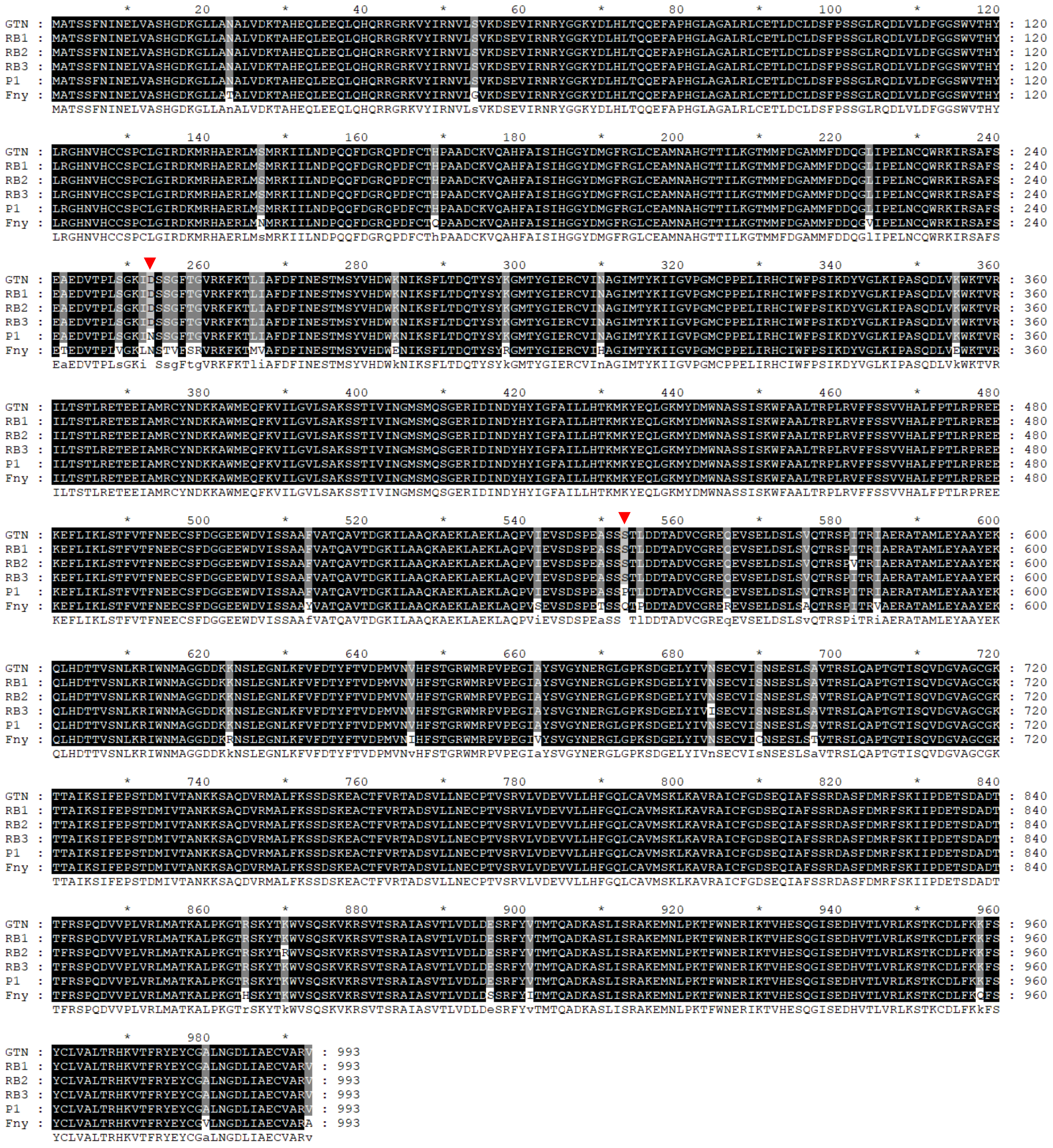

Supplementary Figure S2. Alignment of amino acid sequences of CMV 1a proteins. Sequences were aligned using ClustalW implemented in MEGA X software. Red arrowheads indicate amino acid positions 253 and 553.

Supplementary Figure S3

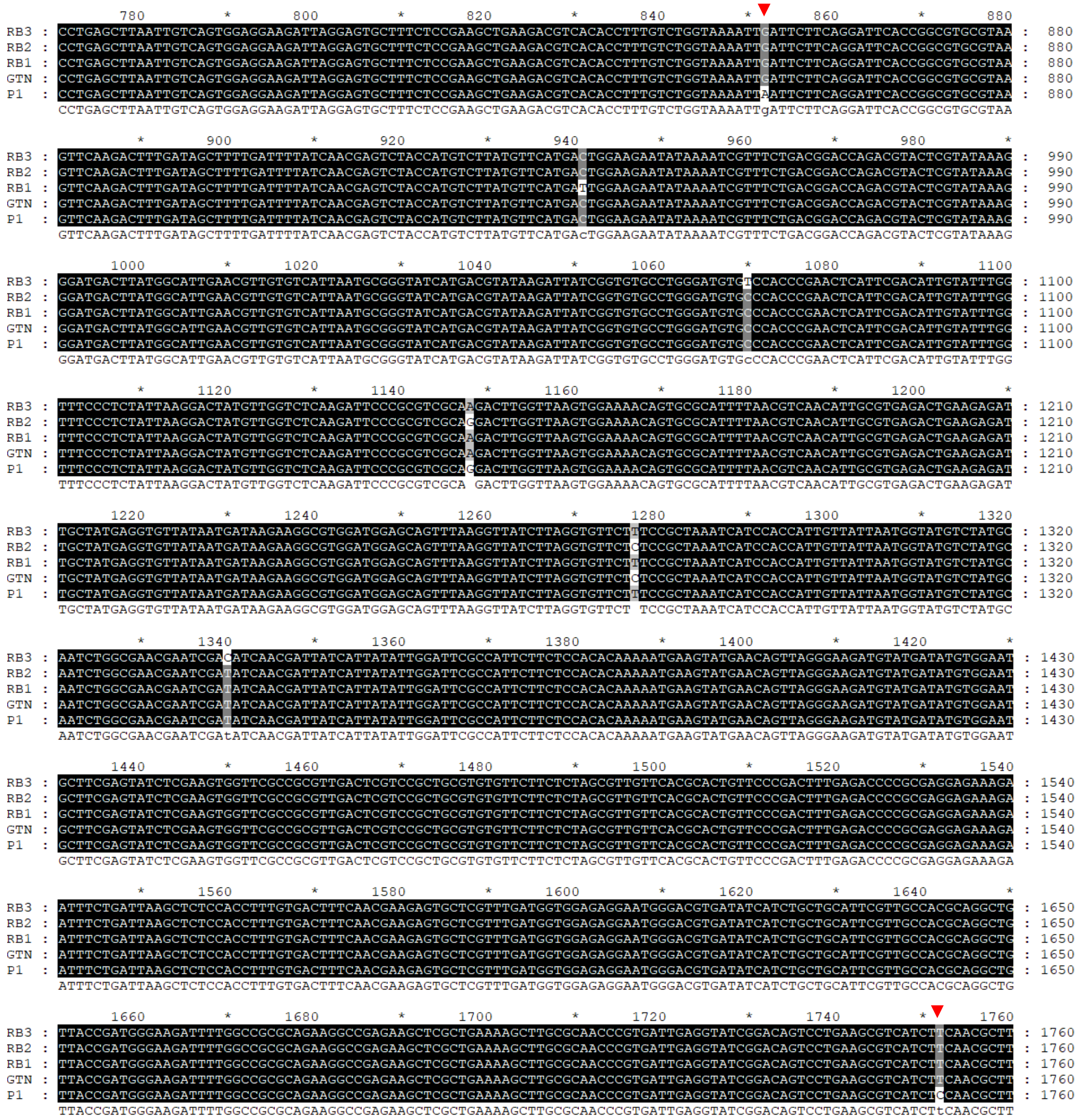

Supplementary Figure S3. Alignment of nucleotide sequences of CMV RNA1. Sequences were aligned using ClustalW implemented in MEGA X software. Red arrowheads indicate nonsynonymous mutations at nucleotide positions 852 and 1752.
